# Long-term care staffs’ experience in facilitating the use of videoconferencing by cognitively impaired long-term care residents during the COVID-19 pandemic: a mixed-methods study

**DOI:** 10.1186/s12913-024-11095-9

**Published:** 2024-05-21

**Authors:** Marie-Soleil Hardy, Chaimaa Fanaki, Camille Savoie, Machelle Wilchesky, Marie-Pierre Gagnon, Maude Laberge, Vincent Couture, André Côté, Clémence Dallaire, Philippe Voyer, Maria Cecilia Gallani, Bernadette Dallaire, Éric Gagnon

**Affiliations:** 1 Faculty of Nursing Science, Université Laval, Québec, QC G1V 0A6 Canada; 2 Department of Family Medicine and Division of Geriatric Medicine, McGill University, Montreal, QC H3S 1Z1 Canada; 3 Faculty of Administration, Université Laval, Québec, QC G1V 0A6 Canada; 4 Faculty of Social Sciences, Université Laval, Québec, QC G1V 0A6 Canada

**Keywords:** Videoconferencing, Long-term care, Cognitive dysfunction, COVID-19

## Abstract

**Background:**

During the COVID-19 pandemic, numerous long-term care (LTC) homes faced restrictions that prevented face-to-face visits. To address this challenge and maintain family connections, many LTC homes facilitated the use of electronic tablets to connect residents with their family caregivers. Our study sought to explore the acceptability of this practice among staff members and managers, focusing on their experiences with facilitating videoconferencing.

**Methods:**

A convergent mixed method research was performed. Qualitative and quantitative data collection through semi-structured interviews to assess the acceptability of videoconferencing in long-term care homes and to explore the characteristics of these settings. Quantitative data on the acceptability of the intervention were collected using a questionnaire developed as part of the project. The study included a convenience sample of 17 staff members and four managers.

**Results:**

Managers described LTC homes’ characteristics, and the way videoconferencing was implemented within their institutions. Affective attitude, burden, ethicality, opportunity costs, perceived effectiveness, and self-efficacy are reported as per the constructs of the Theoretical Framework of Acceptability. The results suggest a favorable acceptability and a positive attitude of managers and staff members toward the use of videoconferencing in long-term care to preserve and promote contact between residents and their family caregivers. However, participants reported some challenges related to the burden and the costs regarding the invested time and staff shortage.

**Conclusions:**

LTC home staff reported a clear understanding of the acceptability and challenges regarding the facilitation of videoconferencing by residents to preserve their contact with family caregivers.

## Introduction

Long-term care (LTC) homes in Canada were heavily shaken by the COVID-19 pandemic crisis. In Canada, around 80% of deaths during the first wave (March through August 2020) were among their residents [1]. To curb the spread of the virus, authorities implemented strict policies, such as social distancing and visit restrictions in LTC homes that inadvertently put residents at increased risk for loneliness [2]. A recent study highlighted how quarantine negatively impacted older adults with cognitive impairment [3], who represent more than 80% of LTC homes’ residents [4]. Existing literature highlights the deleterious effects of COVID-19-related isolation on older adults [3, 5, 6] and consequences for family caregivers [3]. The latter authors report acute deterioration in cognitive and behavioral function among residents during the first wave of the pandemic.

Family caregivers provide emotional and care support for older adults, especially those suffering from chronic and complex health conditions [7]. They play a vital role in supporting their older relatives as care recipients. Their involvement in the older adults’ decision-making has been associated with positive effects for family well-being [8].

To promote the presence of family caregivers despite visit restrictions, we proposed that LTC homes could benefit from technology like videoconferencing, using mainstream communication platforms (e.g., Zoom, Skype, Microsoft Teams) [9]. Technology use, supported by values of empowerment, respect for individuals and their right to self-determination, is considered as being a key factor to enable comprehensive person-centred care [10]. Studies show that the use of electronic devices to promote contact with loved ones significantly reduces feelings of loneliness in seniors, improves physical abilities and vitality, and promotes pain management [11, 12]. To maintain family support, LTC home staff facilitated electronic tablets ‘use by residents to maintain the communication with their caregivers and loved ones, especially during the first waves, where in-person visits were prohibited. This experience brought different challenges and efforts of adaptation for the LTC homes’ staff members who were responsible for integrating these tasks into their work schedule and care routine. However, to our best knowledge, there has been no previous study in Canada that has evaluated the implementation of similar innovative technologies aimed at alleviating negative consequences among cognitively impaired seniors in LTC homes in the context of a pandemic. The aim of this article is to describe the acceptability of videoconferencing facilitation by LTC staff members and managers.

## Methods

### Study design

We conducted a convergent mixed method research, where we collected both quantitative and qualitative data, which were then integrated for the interpretation of the overall results [13]. This study is part of a larger research project aimed at evaluating the implementation process, viability, and acceptability of interventions to support the presence of family caregivers, as well as to examine the effects on residents and their family caregivers and related costs.

### Setting and sample

Four LTC homes in the province of Quebec, Canada participated in our project. The diversity regarding their environment (urban, rural) or their organization status (public, private) enabled us to understand the various factors that can influence the degree and variability of implementation.

With the help of promotional posters and presentations of our research project that were diffused across settings, we have recruited a convenient sample consisting of 17 staff members and 4 managers.

Staff members were approached by the recruited managers and had to:1) be 18 years of age or older; 2) work in the LTC homes for at least 3 months; 3) be a member of the team providing services to residents (nurses, physiotherapists, occupational therapists, specialized educators, recreational activities technicians, etc..). In addition, the participating staff members had to facilitate videoconferencing communication between residents and their family caregivers. Managers had to be involved in the management and the direction of the LTC homes care activities.

### Intervention

Each LTC was required to ensure the weekly occurrence of at least one videoconference meeting between residents and their relatives, over a three-month period. All staff members received written information on strategies for assisting residents that suffered from major neurocognitive problems and their loved ones during a videoconference.

### Data collection methods

Following the instructions of public health limiting access to LTC homes, data collection was done virtually by research assistants from March 2021 to October 2021. Separate and different data collection were done with staff members and managers through an hour-long individual interviews where research assistants took detailed notes in the form of real time verbatim without any audio recording due to the need to produce results within a constrained timeframe to inform our partners and deliver key findings as stipulated by the funding terms. All identifiable data were anonymized to safeguard the privacy of the participants.

Staff members’ qualitative and quantitative data were collected at an interval of three months between (T1) and (T2) for each LTC. Semi-structured individual interviews were conducted by research assistants with all staff members using an interview guide inspired by the Theoretical Framework of Acceptability (TFA) and its six constructs [14] that were presented on Table 1. We did not include the construct “Intervention coherence” of the Sekhon et al. [14] model because it did not apply to our intervention (which is the use of tablets).

**Table 1:** Six TFA Constructs retained and their definitions [14]

Construct	Definition
Affective attitude	How staff members feel about the intervention
Burden	The perceived amount of effort that is required to participate in the intervention
Ethics	The extent to which the intervention is perceived as a good fit with their value system
Opportunity costs	The extent to which benefits, profits, or values must be given up when engaging in the intervention
Perceived effectiveness	The extent to which the intervention is perceived to be likely to achieve its purpose
Self-efficacy	The participant’s confidence that they can perform the behaviour(s) is required to support and participate in the intervention

Interviews were designed to explore staff members’ experiences in assisting residents and caregivers with videoconferencing, as well as the challenges and resources (facilitators) during intervention implementation and use. Interview guides were presented to two partner healthcare professionals before the beginning of data collection. Modifications were made following their comments to facilitate participants’ understanding of the questions.

Quantitative data on the intervention’s acceptability were collected at the same time as the interviews. Staff members rated each of the six TFA constructs (Table 1) on a scale from 0 to 10, where 10 indicates highest level of acceptability of the intervention (except for the constructs pertaining to « Opportunity cost» and « Burden», where the scale was inverted).

Only qualitative data were collected with managers at T1 through a semi-structured individual interview to provide an overall understanding of the philosophy, physical environment, clinical and quality of life programs, work organization, staffing and technology use in each LTC home.

Sociodemographic data from all participants were collected at the beginning of the interview at T1.

### Data analysis

For qualitative data, we have used a deductive approach by building an analytical framework on an excel table based on the six constructs of the TFA. Two research assistants then analyzed data independently. They later met with the principal investigator to reach consensus and validate the themes. Quantitative data were analyzed using IBM SPSS Statistics version 28 software. The sociodemographic and construct data were analyzed descriptively, and frequency distributions, means, and medians were calculated.

Once the qualitative and quantitative data were analyzed, the findings from both approaches (quantitative and qualitative) were merged to observe convergence and divergence between the findings.

### Ethical considerations

The protocol was approved by the CISSS Chaudière-Appalaches Ethics Board (2021–846—ESMO-ESLD). Permission for the research was given by the Direction *Soutien à l’autonomie des personnes âgées – CISSS* Chaudière-Appalaches (QC). All methods were carried out in accordance with relevant guidelines and regulations and Declaration of Helsinki. Due to social restriction measures during the pandemic, the research assistant contacted all participants, read and sent a copy of the consent form by email. All participants provided a verbal informed consent to participate in the research project which was approved by the ethics board.

## Results

First, participants’ sociodemographic characteristics are presented. Next, managers’ interviews results are presented. Finally, staff members’ acceptability of videoconferencing is presented in terms of qualitative and quantitative results.

### Participants’ characteristics

Participant’s sociodemographic characteristics are presented in Table 2. Three out of four LTC home managers were women. The sample represented a wide age range. They were occupying their position for an average of 7.3 years.

**Table 2:** Sociodemographic characteristics of managers and staff members

Characteristics/Variable	Results
**Managers (*n* = 4)**	
Age range	
30 to 39 [no. (%)]	1 (25)
40 to 49 [no. (%)]	1 (25)
50 to 59 [no. (%)]	1 (25)
60 or more [no. (%)]	1 (25)
Gender	
Male [no. (%)]	1 (25)
Female [no. (%)]	3 (75)
Job title	
Director of nursing care [no. (%)]	2 (50)
Manager [no. (%)]	2 (50)
Number of months employed by LTC home [mean ± SD (range)]	87 ± 45.5 (42–144)
**Staff members (*n* = 17)**	
Age (yrs.) [mean ± SD (range)]	37.1 ± 12.5 (19–57)
Gender	
Male [no. (%)]	2 (11,8)
Female [no. (%)]	15 (88.2)
Job title	
Specialized educators [no. (%)]	5 (30)
Recreational activities technicians [no. (%)]	3 (17.6)
Nurse [no. (%)]	3 (17.6)
Other [no. (%)]	6 (35.3)
Number of months employed by LTC home [mean ± SD (range)]	105.2 ± 130.4 (0.75–402)
Number of hours worked per week [mean ± SD (range)]	33.0 ± 6.3 (15–40)
Work Shift	
Daytime [no. (%)]	16 (94.1)
Variable [no. (%)]	1 (5.9)

17 staff members participated at T1 and 12 at T2 since five members left their position during the study. Most staff members were female, with a mean age of 37.1 years. Participants occupied diverse job positions and had been working in their LTC home for an average of 8.8 years. The majority worked full time during the day shift. The “other” category of job title includes, but is not limited to, trainees, recreational services coordinator, and animation assistants.

### Description of LTC home by managers

All managers described their LTC home as a personalized living environment, where they tried to match with each resident’s past lifestyle as closely as possible. “*We proceed with an approach where the resident has to feel like they are at home*”. (Etab1_GEST01). All managers placed great importance on the presence of the family and on maintaining a good relationship with them: “*Families are very present in the decisions we make. We are a transparent environment. We consult family members for all kinds of situations; we are truly in partnership in the decision-making process.*” (Etab4_GEST01). To this end, several spaces had been set up in LTC homes for meetings between residents and their loved ones, such as the residents’ rooms, common lounges, dining rooms, corridors, and outdoor courtyards. Some facility spaces were restricted due to COVID-19 to limit the spread of the virus.

Managers also highlighted the effect of the presence of the family in their settings on residents’ well-being. A manager explained how the lack of visits took a toll on residents: “*It had a very big impact… We saw impacts on the health and mental state of the residents*.” (Etab4_GEST01).

To maintain communication through videoconferencing, managers reported that they adapted the care schedule as much as possible to the residents’ needs, habits, and routines. Family caregivers were aware of the care schedule and could plan the meeting time, beforehand. While different staff managed meetings and communications, including nurses, nursing assistants, and special educators, recreational technicians were mostly in charge supervising videoconferencing. Most managers reported that they had to solicit the aid of additional staff members to keep up with the increased demand for videoconference meetings during the pandemic.

The assigned staff were responsible for planning: “*Recreational technicians will plan the meeting, they will send the teams link to the relative by email, they will do a test in the office with the relative and to see if it works*” (Etab2_GEST01). One manager specified that some calls were initiated spontaneously if residents had a particular or urgent need. Before meetings, staff members also prepared residents and made sure that they were in a suitable setting to ensure smooth communication: “*We’re going to prepare the resident, so, proceed with the hygiene, make sure the resident has their hair/makeup done that the resident is in a well-lit environment, that the resident is in a nice place to make the call*.” (Etab4_GEST01). Staff members also ensured the support and the good progress of the meetings: “*It is the recreational technicians who will hold the tablet, and make sure that the camera remains in front of the user. The staff member will also sometimes intervene in the meeting, for example, if the resident does not speak. He will sometimes speak with the caregivers to give news*.” (Etab2_GEST01).

In addition to tablets, other technological equipment such as bigger screens were used in some settings to project the image during the call: “*We have televisions, which we can use to broadcast the video call on a larger screen than the tablet. This is a very popular trick to accommodate our residents, especially those with severe cognitive impairments, as residents sometimes have difficulty focusing well on the small screen of the tablet*” (Etab1_GEST01). Unfortunately, not all settings (one out of four) had access to an Internet network, which was limiting. Some LTC homes also bought a telephone handset to connect it to the tablet. The handset helped some residents with hearing problems to follow the conversation, or for the sake of familiarity, making them feel like they were talking on the phone.

### Staff members’ acceptability of videoconferencing

**Fig. 1:**
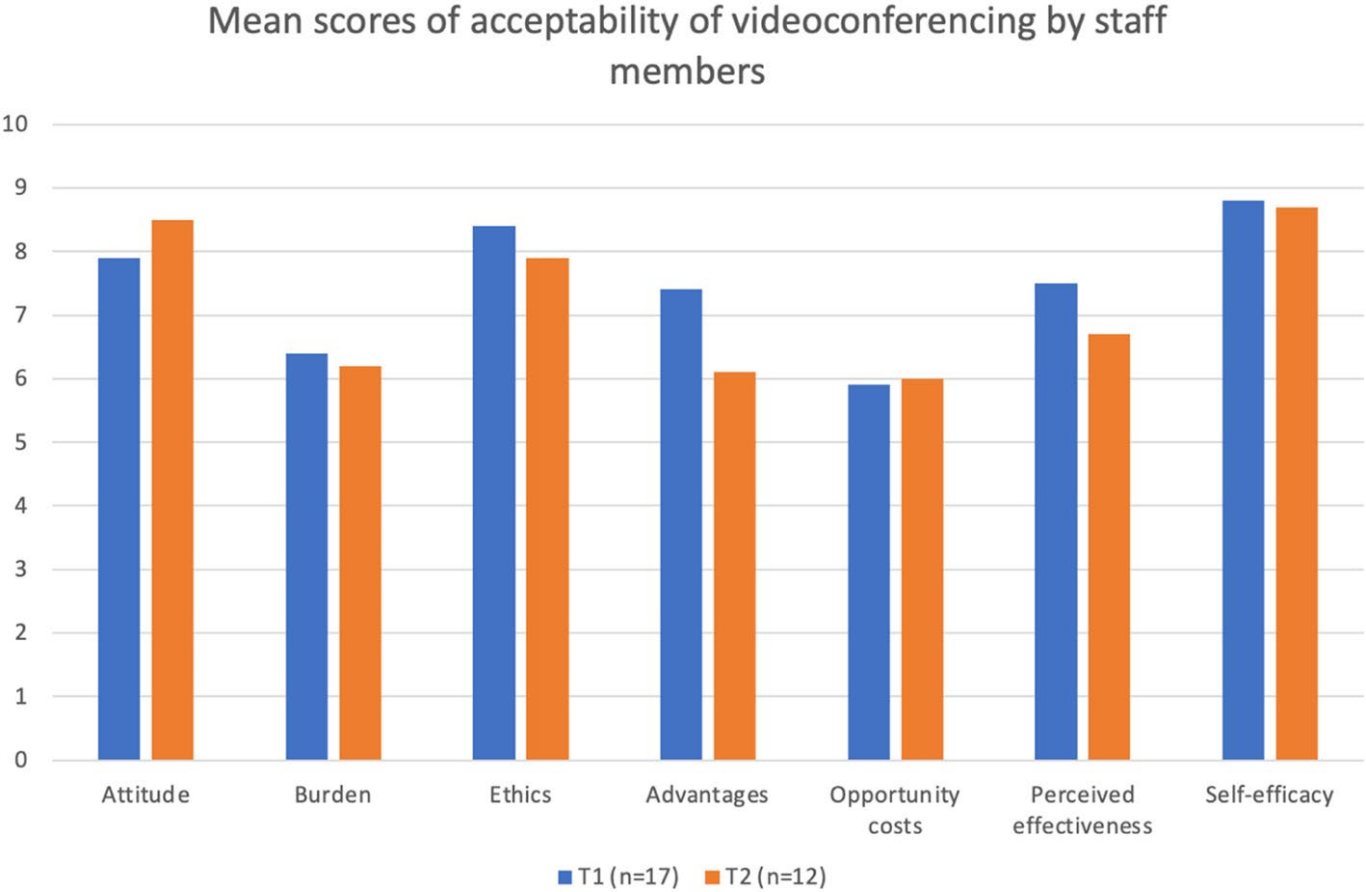
Mean scores of acceptability of videoconferencing by staff members

Quantitative results of staff members’ acceptability of videoconferencing are shown in Fig. 1. The scores obtained showed stability between T1 and T2.

Qualitative and quantitative findings are presented below for each TFA construct.

### Affective attitude

Staff members showed a positive attitude toward the use of videoconferencing to preserve contact between residents and their caregivers (mean score around 8 out of 10). Most staff members had a positive experience when assisting residents during their videoconferences with families. Videoconferencing helped to keep residents connected to their loved ones, to increase interaction between them, and to create unique moments. For example, one staff member described the caregivers as their clients as much as the residents themselves “*In general, I think it’s great to be able to do this for the families and for the residents. Sometimes I feel like I’m doing it more for the residents, sometimes I’m doing it more for the family, but I consider both to be my clients. I realized that for the families, to see them, it makes a difference. It’s important for people because it allows them to keep a connection*.” (Etab1_PROF03).

On the other hand, some staff members mentioned that the resident’s mental state affected how the videoconferences were conducted. For instance, certain residents faced challenges in recognizing their family members, some became upset during video calls as they couldn't physically touch their loved ones through the screen. A few residents would even fall asleep during the calls. To this end, most staff members perceived that videoconferencing was beneficial primarily for families.

### Burden

Almost all staff members reported that assisting residents during videoconference required a lot of effort (scores of 6,4 and 6,2 out of 10). Most residents required continual assistance due to their cognitive and other impairments. One staff member explained: “*For people who are more independent, it doesn’t take much effort because you just have to set up the tablet. However, for people who are less independent, you have to set them up, be with them during the meeting, stimulate them, which is more demanding. You have to keep the resident awake. We are a communication support*.” (Etab4_PROF04). Moreover, some staff and family caregivers weren’t comfortable using videoconference platforms or tablets. For example, one staff member added: “*In terms of the technology, we had to adapt to that and show the family how to do it. We had to be patient and accommodating. Sometimes it took two people. It took a lot of energy*.” (Etab1_PROF04). Eventually, it caused an overload of work by adding extra tasks to the staff’s schedules: “*It still fits into our schedule but it’s too busy too so we’re tight, our daily routine didn’t change but we were adding more tasks each time!*” (Etab1_PROF01). Sometimes this could lead to cutting other activities, which some of them deemed more useful for the stimulation of many residents.

### Ethicality

The intervention was also a good fit with their value system (scores of 8.4 and 7.9 on ethics variable). However, some staff members mentioned that this mode of communication did not match with their values at all, as they preferred face-to-face visits. Several participants reported the lack of privacy: “*For sure, the in-person visits were better because the families stayed with the residents longer without us being there, so they had more privacy and direct interaction with the residents*” (Etab5_PROF02). To this end, several staff members characterized videoconferencing as a default means of communication considering the restrictions on visitations, related to the COVID-19 pandemic: “*Sure it would be better with face-to-face visits, but we can’t, so it’s better than nothing. It’s a good alternative*” (Etab1_PROF06). Finally, a few staff members mentioned the importance of videoconferencing, as it was important for them to maintain relationships and contact between residents and their loved ones: “*I can understand that a family feels helpless when they can’t see them in person. You can’t be against this means of communication when you see the reaction of the relatives*” (Etab5_PROF02).

### Opportunity costs

Average cost scores are 5,9 and 6,0, reflecting a certain amount of costs related to videoconferencing across different settings. Indeed, the analysis of the staff interviews showed that several adjustments were required for the integration of videoconferencing within work routines. First, it required scheduling adjustments to fit it into the care schedule, as one participant noted: “*It takes a lot of time (…). So, if we have a lot of requests during the day, I’ll be forced to focus on the calls and not on my other duties.*” (Etab5_PROF02). In addition, it was difficult to balance the time spent on videoconferencing with that spent on other leisure activities: “*It was the main activity, we dropped everything else. So, in this context where that’s all we do, well, it fits in well. It was the only thing to do. Now that we’re offering other activities again, it’s harder to integrate*.” (Etab5_PROF01). Indeed, adding videoconferences into the care schedule was reported as a challenge by several staff members: “*We have time slots that we try to keep up with, but sometimes these meetings can play on the actual planning to devote more time for meetings.*” (Etab4_PROF03).

In doing so, most participants reported that integrating videoconferencing via tablets required a notable time investment. “*It takes a lot of time. The residents are not able to do it on their own. […] You must do everything. It’s everything from planning the call to getting off the phone. Some residents I leave alone in the room with the tablet during the call, but sometimes it’s hard. There are some that tap the tablet, so they end up with windows and pictures*”. (Etab1_PROF03). Other staff members reported that the time investment depended on the resident’s ability to stay alone during videoconferences: “*It's variable, it depends, for some you have to be with them all the time and some I can leave once the zoom is set up. It’s different from one resident to another*” (Etab2_PROF01).

### Perceived effectiveness

Videoconferencing seems to achieve its purpose to preserve contact, with a score around 7.0 in T1 and T2. According to participants, videoconferencing keeps residents connected to their loved ones, especially when family caregivers were restricted from visiting residents. One staff member said: “*I think we hit that target. You have to put it in the context that the loved one can’t see [the residents] so the video was a solution to keep the connection and communication between the residents and their loved ones. I’ve seen a lot of things exchanged and shared through these communications, like showing newborns, recipes and stories being shared, *etc*. For people with severe [cognitive] disorders, even though we can’t assess the situation, we still see reactions and emotions come out during these calls.*” (Etab4_PROF01). For some residents, videoconferencing helped to maintain their psychological health: “*Everything that was mediated, the anxiety rose for the most lucid. The more affected ones didn’t understand why there was no more activity, they felt abandoned. So, videoconferencing was a stimulating activity. I think it helped them with their psychological health. That’s a problem we had for a while; morale was low among our residents.*” (Etab5_PROF01). It also helped to stimulate even the most cognitively impaired residents: “*Another lady this morning, she was asleep, so I sat down next to her and called her son, so I put him on the speaker. When the son started talking, the lady woke up and started making sounds. I think that with what I’ve seen since we started doing this, no matter what stage the patient is in, it awakens the residents, it touches something, that’s for sure.*” (Etab1_PROF03).

On the other hand, a few participants reported that videoconferencing remained a default means of communication, compared to in-person visits. One staff member said: “*Face-to-face interactions are more beneficial than the other means, you can touch them for example, while it’s not possible using the videoconferencing*.” (Etab1_PROF01). Finally, some participants reported that it was difficult to judge the usefulness of videoconferencing in maintaining quality contact and breaking isolation because residents have severe cognitive impairment. One participant said: “*It’s because of their neurocognitive impairment. I find that these people have less or no interest in looking at a camera, sometimes they don’t even understand. So, it takes a physical presence to be able to be stimulated. For other residents who have no or little [cognitive] impairment, these interventions are more beneficial because they are always happy to talk to their loved ones.”* (Etab5_PROF04).

### Self-efficacy

All staff members interviewed reported feeling confident and competent to assist residents with their communication with the tablet, with a score around 9 out of 10 in self-efficacy. In addition, professionals reported that their sense of competence improved over time: “*At first, I didn’t feel competent at all. It got better over time.*” (Etab1_PROF04).

## Discussion

The present study aimed to describe the acceptability of videoconference by the staff members and managers of four LTC homes. Across different LTC homes, managers and staff members acknowledged the importance of the family’s presence in their settings and their role in the residents’ lives and well-being. They consider the residents’ families to be as much as their clients as the residents themselves. They involve them in decision-making and in their relative’s care. In Canada, family caregivers spend many hours caring for their relatives in LTC homes, especially those suffering from cognitive problems. They promote and provide emotional support, social engagement, advocate for their relatives, oversee their care and contribute resources and ideas to the LTC home community [15]. Following the restriction of visits during the pandemic, managers and staff members noticed how the absence of family caregivers impacted the mental and physical health of their residents. To maintain communication and to counter the potential adverse effects that these restrictions might inflict, LTC homes turned to videoconferencing as an alternative strategy. Social isolation was associated with negative deleterious outcomes, including an increase in depression, cognitive decline, and behavioural symptoms of dementia [16]. Several studies have shown how the withdrawal of different social activities and the restrictive measures that were put in place created confusion among these residents [17, 18]. These results were similarly reported by our participating LTC home managers and staff in our study and applies particularly to residents with severe cognitive impairment. Moreover, videoconference was generally perceived positively by all the staff and managers, similarly to our previously published findings that report on acceptability by caregivers and residents [9]. The videoconferencing intervention purpose fit within the culture and philosophy of LTC homes that strive to embrace a person-centred care model that prioritizes personhood and quality of life, by creating and supporting collaborative relationships among workers, family caregivers, and residents. Personalizing the means of communication and their frequency according to family needs and resident characteristics could be considered to ensure adherence and the desired effects.

The use of tablets for videoconferencing was newly introduced to these LTC homes during the pandemic. Most sites, however, were not ready or equipped to properly organize and manage their implementation. First, most settings had only few tablets, which wasn’t practical to serve the high demand for videoconferences. They didn’t have access to a public Wi-Fi network, so staff could not easily connect and commute across different rooms and areas. Moreover, the reported absence of practice standards and guidelines for such interventions in long-term care left the whole process arbitrary (i.e., up to each care home), which explains the variability in the implementation approach across sites. For example, different sites assigned different staff members to organize videoconference sessions with families and to support residents when needed. However, data from managers and staff show that this intervention is interdisciplinary and involves multiple activities to ensure a positive experience for everyone. As such, a variety of healthcare professionals were involved in the management of videoconferences (e.g., nurses, recreational activities technicians, special educators). The videoconference significantly mobilizes the staff since several steps are necessary for videoconference success. For example, the staff must plan the meeting with the family beforehand, they must dress the resident, take him/her to a quiet place, support the family in technical difficulties, assist the resident during the videoconference, etc. In doing so, videoconferencing represents a significant additional workload for staff; much more than in-person visits, for which staff have no special preparation. On the other hand, videoconferencing requires less effort for caregivers since they connect remotely for a few minutes from their home. It is not surprising, therefore, that when we compare the ‘burden’ scores of family caregivers from our previous study [9] and staff, we see that the score is higher for staff than for family caregivers.

Managers mentioned that their sites have adequately adapted and personalized work routines and care schedules to allow for smooth integration of videoconferencing. Staff members, however, reported a somewhat different reality. In fact, integrating this new role within their usual daily routine was challenging, especially in the context of staff shortage and turnover faced in this setting during the pandemic. Staffing shortages are not new to LTC homes, as this was already a challenging reality before the pandemic [17, 18]. Thus, to support tablet use and organize videoconference meetings, staff members had to divide their work time between all residents, hindering or cancelling other activities that were perceived as being important and beneficial for the stimulation of those who were cognitively impaired. To be able to promote stimulation and increase contact rates, LTC homes need to be equipped with technologies and environments that are more adapted to the needs of the elderly with neurocognitive disorders.

### Limitations

While conducting the study during the COVID-19 pandemic was a strength, it also presented challenges, especially due to the restrictions in long-term care settings at that time. The small sample size in this study may impact the external validity of the quantitative data. The generalizability of the findings is limited due to the small sample size. Additionally, real-time transcription of qualitative data, instead of recording, could potentially raise issues regarding its credibility and confirmability. However, the triangulation of qualitative and quantitative data enhances the rigor of the study and enables valid conclusions to be drawn.

## Conclusions

The results suggest a favorable acceptability and a positive attitude toward the use of videoconferencing in long-term care to preserve and promote contact between residents and their family caregivers. Videoconference implementation in these settings faced some challenges, both for staff members and for managers, especially in terms of the burden and opportunity costs regarding the invested time and staff shortage that must be tackled to achieve a sustained implementation. Videoconferencing is a complementary option to face to face visits. LTC homes should consider the use of such technology since it creates an opportunity to stimulate residents with major neurocognitive problems and increase their contact with loved ones.

## Data Availability

The datasets generated and/or analyzed during the current study are not publicly available due to ethical concerns and restrictions imposed by the ethics committee, and prior agreements with participants, but are available from the corresponding author on reasonable request.

## Abbreviations

LTC: Long term care
TFA: Theoretical Framework of Acceptability
